# In Vitro and In Vivo Assessments of Newly Isolated N4-like Bacteriophage against ST45 K62 Capsular-Type Carbapenem-Resistant *Klebsiella pneumoniae*: vB_kpnP_KPYAP-1

**DOI:** 10.3390/ijms25179595

**Published:** 2024-09-04

**Authors:** Shanmuga Priya Natarajan, Soon-Hian Teh, Ling-Chun Lin, Nien-Tsung Lin

**Affiliations:** 1 Master Program in Biomedical Sciences, School of Medicine, Tzu Chi University, No. 701, Sec. 3, Zhongyang Rd., Hualien 97004, Taiwan; 109329107@gms.tcu.edu.tw; 2 Division of Infectious Diseases, Department of Internal Medicine, Hualien Tzu Chi Hospital, Buddhist Tzu Chi Medical Foundation, No. 707, Sec. 3, Zhongyang Rd., Hualien 97004, Taiwan; jimmyteh@tzuchi.com.tw

**Keywords:** carbapenemase-producing *Klebsiella pneumoniae*, carbapenemase genes, capsular type, N4-like phage, phage therapy

## Abstract

The rise of carbapenem-resistant *Klebsiella pneumoniae* (CRKP) presents a significant global challenge in clinical and healthcare settings, severely limiting treatment options. This study aimed to utilize a bacteriophage as an alternative therapy against carbapenem-resistant *K. pneumoniae*. A novel lytic N4-like *Klebsiella* phage, vB_kpnP_KPYAP-1 (KPYAP-1), was isolated from sewage. It demonstrated efficacy against the K62 serotype polysaccharide capsule of *bla*_OXA-48_-producing *K. pneumoniae*. KPYAP-1 forms small, clear plaques, has a latent period of 20 min, and reaches a growth plateau at 35 min, with a burst size of 473 plaque-forming units (PFUs) per infected cell. Phylogenetic analysis places KPYAP-1 in the *Schitoviridae* family, *Enquatrovirinae* subfamily, and *Kaypoctavirus* genus. KPYAP-1 employs an N4-like direct terminal repeat mechanism for genome packaging and encodes a large virion-encapsulated RNA polymerase. It lacks integrase or repressor genes, antibiotic resistance genes, bacterial virulence factors, and toxins, ensuring its safety for therapeutic use. Comparative genome analysis revealed that the KPYAP-1 genome is most similar to the KP8 genome, yet differs in tail fiber protein, indicating variations in host recognition. In a zebrafish infection model, KPYAP-1 significantly improved the survival rate of infected fish by 92% at a multiplicity of infection (MOI) of 10, demonstrating its potential for in vivo treatment. These results highlight KPYAP-1 as a promising candidate for developing phage-based therapies targeting carbapenemase-producing *K. pneumoniae*.

## 1. Introduction

*Klebsiella pneumoniae* is a Gram-negative bacterium that is non-motile and encapsulated, known for being an opportunistic pathogen. It is linked to a wide range of infections, both in hospital and community settings, such as soft tissue infections, meningitis, liver abscesses, urinary tract infections, surgical site infections, catheter-related infections, pneumonia, and septicemia. These infections predominantly occur in individuals with compromised immune systems [1]. These infections are commonly known for their swift onset and resistance to multiple drugs, including third-generation cephalosporins and/or carbapenems [2]. Extended-spectrum β-lactamase-producing strains of *K. pneumoniae* have become a worldwide threat, posing serious risks to hospitalized patients and increasingly affecting otherwise healthy individuals in the community [3]. The rise and dissemination of carbapenem-resistant *K. pneumoniae* (CRKP) strains in hospitals is a concerning development associated with a high mortality rate of 40–70% [4]. As a result, CRKP are critical nosocomial pathogens responsible for severe and life-threatening infections, particularly in intensive care units, raising significant concern among healthcare providers [5]. *K. pneumoniae* cells have an outermost layer comprising polysaccharide capsules (CPSs), which act as a physical barrier against host immunity and antibiotics, form biofilms, and confer resistance to antibiotics, and, hence, are a major virulence factor of the bacterium [3]. There is a tremendous amount of structural variability, with over 130 capsular types (K-types) among *K. pneumoniae* CPS, and K1 and K2 types are the most virulent [6,7]. Specific genes in the capsular synthesis cluster are associated with each K-type. The cps cluster displays a mosaic structure, containing a conserved group of six genes (*galF*, *orf2*, *wzi*, *wza*, *wzb*, and *wzc*) at its 5′ end, which can be employed for molecular serotyping of *K. pneumoniae* strains [8,9]. In Taiwan, alongside the prevalent K1 and K2 serotypes, the K20, K54, and K62 serotypes are also present. These serotypes harbor additional virulence factors, particularly in patients with bacteremia, suggesting a potential link to increased invasiveness [10]. Additionally, antibacterial susceptibility tests reveal that K1, K2, K20, and K54 are generally sensitive to most antibiotics, whereas K62 shows higher resistance compared to both other typeable and non-typeable strains [10].

As noted earlier, the World Health Organization (WHO) considers this a priority antimicrobial-resistant (AMR) pathogen on a global scale, highlighting the urgent need for new control strategies [11]. Bacteriophage (phage) therapy is an alternative strategy to treat carbapenem-resistant *K. pneumoniae* strains [12]. Multiple promising *K. pneumoniae* phages have been identified, with studies showing their potential therapeutic effectiveness in animal infection models [13,14,15,16,17,18,19,20]. Several case studies have also used anti-*Klebsiella* phages as a last-resort treatment for human drug-resistant infections [21,22]. However, significant diversity exists among pathogenic bacteria and phages. Given the numerous K-types of *K. pneumoniae*, this study aimed to isolate new phages targeting the K62 capsular strain, which has emerged as one of the five most prevalent serotypes in Taiwan [10]. This strain has also demonstrated significant resistance to multiple antibiotics over the course of a two-decade seroepidemiological study, highlighting the need to carefully analyze and address the challenges posed by K62 strains in Taiwan. Moreover, to date, only one phage, SH-KP2492, has been reported to target strains of this serotype [23]. Here, we isolated a novel lytic N4-like phage vB_KpnP_KPYAP-1 (KPYAP-1) from a waste water source in Taiwan. This phage is specific to carbapenemase-producing *K. pneumonia* of capsule type K62. Furthermore, the whole genome of KPYAP-1 was sequenced, and the genetic background was defined by bioinformatics analysis and proposed taxonomy. Considering its potential value in anti-*K. pneumoniae* phage therapy, we evaluated its therapeutic efficacy during clinical CRKP strain infection using a zebrafish model.

## 2. Results

### 2.1. Characteristics of K. pneumoniae Clinical Isolated Strains

Forty-seven clinical isolates sourced from Tzu Chi Hospital underwent 16S rDNA amplifications via colony PCR, followed by Sanger sequencing to ascertain their identity as *K. pneumoniae*. Subsequently, their inhibitory zones against imipenem and meropenem were determined to be ≤19 mm utilizing the Kirby–Bauer disk diffusion method, indicating their classification as carbapenem-resistant strains. Further validation through CHROMagar plate testing revealed the consistent production of carbapenemase (carbapenem-hydrolyzing β-lactamases) across all isolates. Carbapenemases are classified into molecular classes A, B, and D β-lactamases. Classes A and D consist of β-lactamases with serine at their active sites, whereas class B β-lactamases are metalloenzymes featuring zinc in their active sites [24]. Among them, the class D carbapenemase *OXA-48* is particularly worrisome due to the challenges in detecting it and its link to treatment failures. Hence, we examined the prevalence of the *OXA-48* gene by polymerase chain reaction (PCR) among these isolates. Based on the PCR results, 68% (32/47) of the isolates carried the *OXA-48* gene. One of the 32 isolates with the *OXA-48* gene (Kp20) was used as a host to isolate the phage KPYAP-1. This strain belongs to the ST45 clonal group with the K62 capsular serotype, the *wzi* allele 149, and the O2a lipopolysaccharide (LPS) antigen.

### 2.2. Isolation and Characterization of Phage vB_KpnP_KPYAP-1 (KPYAP-1)

**Figure 1:**
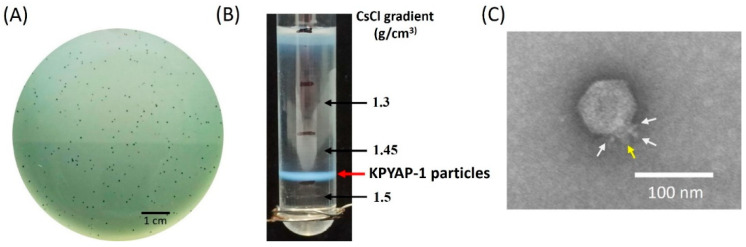
Phage KPYAP-1 morphology and buoyant density. (**A**) The plaque morphology of phage KPYAP-1 as observed on a bacterial overlay plate. (**B**) Analysis using a CsCl gradient revealed that the buoyant density of phage KPYAP-1 falls below 1.45 g/cm^3^. (**C**) An electron micrograph of KPYAP-1 virions. The CsCl-purified viral particles were stained with 2% uranyl acetate and examined at a 120,000× magnification. The phage particles display a short tail (indicated by the yellow arrow) surrounded by fibers (indicated by the white arrow) and are categorized as podophages.

The phage KPYAP-1 was isolated from Tzu Chi Hospital sewage by co-cultivating it with the host K62-type *K. pneumoniae* strain Kp20. This was done using spot tests with Kp20 as the bacterial lawn. The phage formed small, visually similar clear plaques about 1–1.5 mm in diameter, without a surrounding halo, on a lawn of the host strain Kp20 (Figure 1A). KPYAP-1 was enriched through repeated plaque purification, resulting in a stock of 10^11^ plaque-forming units (PFUs)/mL. The stock was then subjected to ultracentrifugation in a discontinuous cesium chloride (CsCl) gradient with densities of 1.5, 1.45, and 1.3 g/cm^3^. Phage particles were found to band in the 1.45 g/cm^3^ block (Figure 1B). Transmission electron microscopy (TEM) revealed that the phage had an icosahedral head measuring 75 ± 5 nm in diameter and a short tail measuring 16 ± 2 nm in length, indicating that it belonged to the podophage family (Figure 1C). Testing on 80 *Enterobacteria* strains, including 47 *Klebsiella* strains, showed that phage KPYAP-1 was specific to *K. pneumoniae* Kp20.

### 2.3. Biological Properties of KPYAP-1

**Figure 2:**
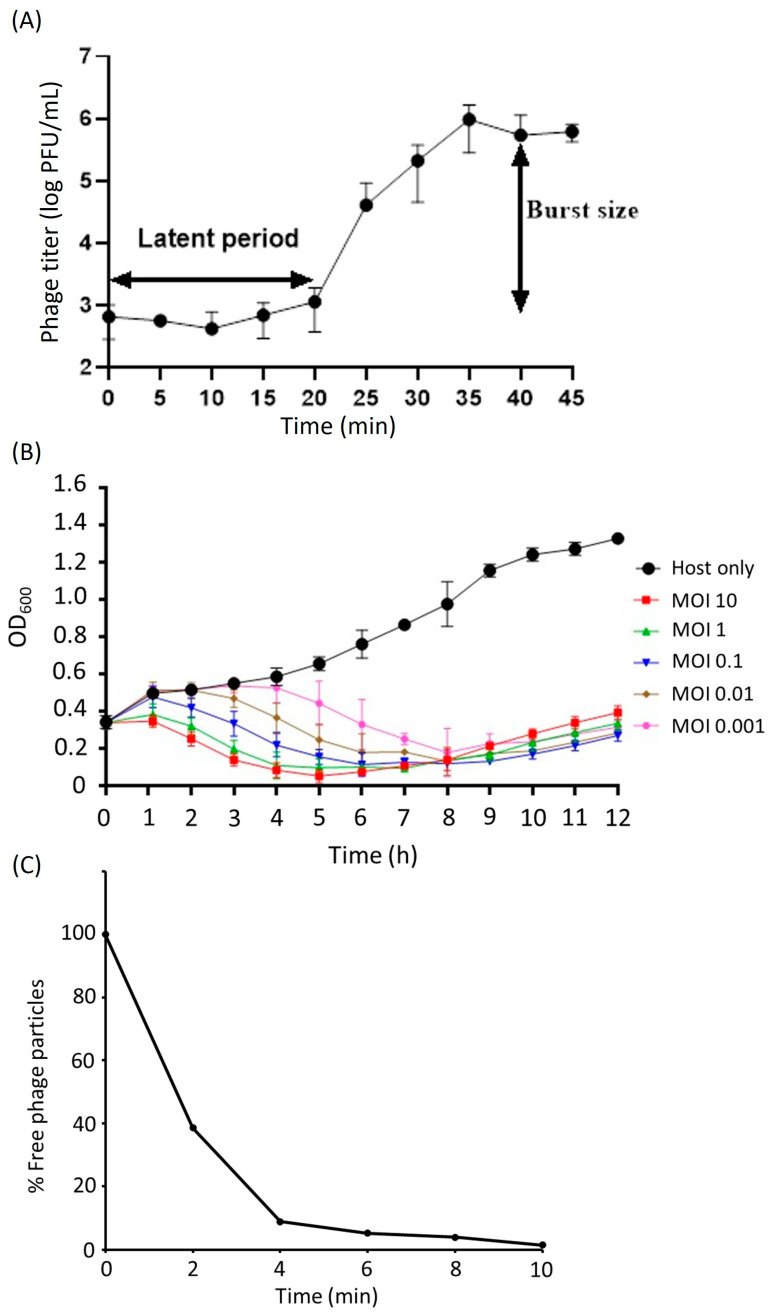
One-step growth, bacteriolytic activity, and adsorption analysis of KPYAP-1. (**A**) When KPYAP-1 was grown on Kp20, it showed a latent period of about 20 min, with a burst size of around 473 plaque-forming units (PFUs) per infected cell. Here, L represents the latent period, and B represents the burst size. (**B**) Bacteriolytic activity of KPYAP-1 against Kp20 was tested at different multiplicities of infection (MOIs). (**C**) Adsorption analysis revealed that approximately 90% of KPYAP-1 particles were attached to Kp20 cells within 4 min, and nearly 99% were adsorbed after 10 min. All experiments were conducted in triplicate.

The results of a one-step growth assay revealed a quick 20 min latent period. The burst size of the phage was estimated to be around 473 PFUs per infected cell. This was determined by dividing the average number of phage particles released (5.4 × 10^5^ PFU/mL) by the average number of phage particles that initially infected the bacterial cells during the latent period (1.14 × 10^3^ PFU/mL) (Figure 2A).

The multistep bacterial killing curve for phage KPYAP-1 showed a significant reduction in OD_600_ value within 8 h post-infection, with the OD_600_ dropping to around 0.1 regardless of the MOI, while the control group containing only the host reached nearly 1 (Figure 2B). However, after 8 h, the OD_600_ gradually increased, likely due to the emergence of phage-resistant strains.

To determine the attachment efficacy of KPYAP-1 to Kp20, the adsorption of phage was calculated at different time points. Nearly 61.5% of the phage was adsorbed to Kp20 within 2 min and almost 99% was adsorbed at 10 min (Figure 2C).

**Figure 3:**
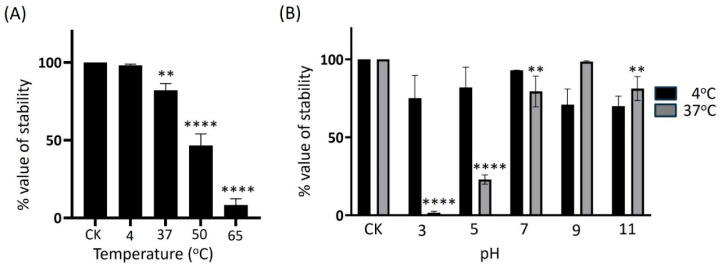
Biophysical stability of KPYAP-1. (**A**) Thermal stability: the thermal stability of KPYAP-1 was examined by incubating the phages at various temperatures for 1 h. (**B**) pH stability: similarly, the pH stability was assessed by incubating the phages at different pH values for 1 h. The experiments focused on the plaque-forming ability of the phages on the host lawn to evaluate their infectious activity compared to the control under the specified conditions. Results are shown as the ratio of the phage titer after testing to the original titer (CK). Significant differences are indicated by asterisks (** *p* ≤ 0.01; **** *p* ≤ 0.0001).

To determine the best storage conditions for preparing adequate phage preparations, we evaluated the infectious stability of KPYAP-1 by exposing it to external factors such as pH levels, temperature variations, and long-term storage. Following a 1 h incubation period at 4 °C, the titer of the KPYAP-1 phage exhibited no significant alteration compared to the initial loading concentration. However, a slight decrease was observed at 37 °C, and a significant reduction to 50% was noted at 50 °C. At 65 °C, only 8% of the activity remained, indicating that KPYAP-1 is sensitive to higher temperatures and is considered thermolabile (Figure 3A).

To evaluate the stability of the phage at different pHs, KPYAP-1 was incubated with sodium chloride–magnesium sulfate (SM) buffer at different pHs at 37 °C or 4 °C for 1 h, and the infective activity of the phage was determined. The infectious activity of KPYAP-1 was stable at pH 9 at 37 °C or 4 °C. However, the infectivity of phages was significantly reduced at pHs 3 and 5 when stored at 37 °C (*p* < 0.0001), and was reduced at neutral pH 7 and alkaline pH 11 (*p* < 0.01). There was no significant difference in the infectivity of KPYAP-1 when stored at 4 °C, regardless of the pH preservation solution (Figure 3B).

In order to evaluate the enduring storage capability of KPYAP-1, phage suspensions were preserved under two conditions: at 4 °C in SM buffer and at −80 °C in SM buffer supplemented with 50% glycerol. Over a period of 12 months, phage titers were assessed every 3 months. The infectious potency of the phage remained consistent throughout the duration of the study, suggesting that both 4 °C and −80 °C storage methods adequately preserved the infectivity stability of KPYAP-1 for at least one year.

### 2.4. Genome Size Determination and Restriction Analysis of Phage DNA

**Figure 4:**
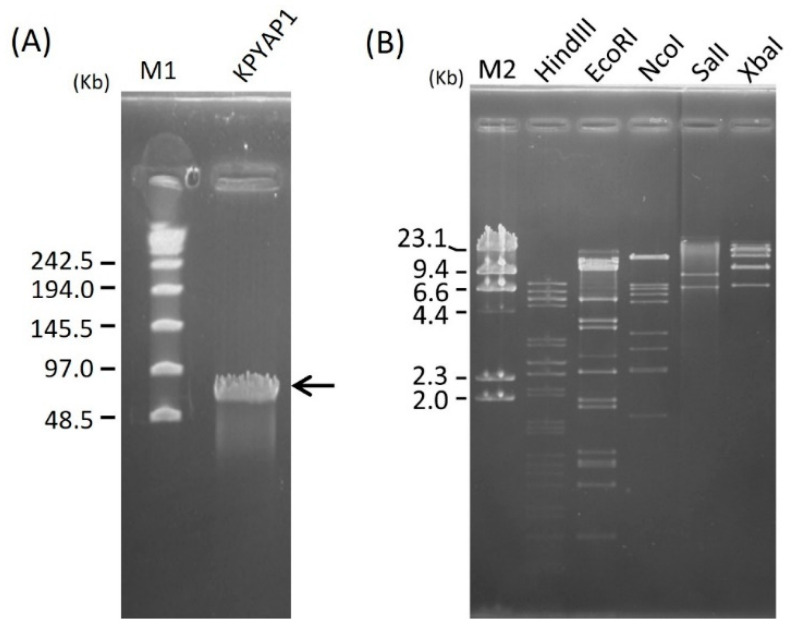
The genome size and restriction analysis of phage KPYAP-1. The estimated size of the KPYAP-1 genome (black arrow) (**A**), and restriction patterns (**B**) were determined by pulse field gel electrophoresis. Lane M1 is the λ PFG ladder; M2 is the λ DNA digested by *Hind*III (New England BioLabs, MA, USA).

To ascertain the size of the KPYAP-1 phage DNA, it underwent analysis via pulsed-field gel electrophoresis (PFGE), revealing an approximate size of 70 Kbp (Figure 4A). Subsequently, the phage DNA’s restriction patterns were examined through PFGE following digestion with *Hind*III, *Eco*RI, *Nco*I, *Sal*I, and *Xba*I. Multiple bands were observed on the gel (Figure 4B), confirming that KPYAP-1 is a double-stranded DNA phage susceptible to digestion by restriction enzymes.

### 2.5. Genome Analysis and Annotation

Whole genome sequencing revealed that KPYAP-1 has a linear double-stranded DNA genome of 73,108 base pairs (bps), with direct terminal repeats of 404 bps at each end. The genome also includes four tRNA genes and a GC content of 44.14%, lower than the average GC content of 57.1% found in *K. pneumoniae*. A BLASTn analysis showed that the genome of KPYAP-1 is closely related to *Klebsiella* phage KP8 (MG 922974), an N4-like podophage, with 88% coverage and 95% similarity. Further examination using PhageLeads [25] confirmed that KPYAP-1 lacks genes linked to temperate or lysogenic life cycles and those related to virulence and antibiotic resistance, indicating a strictly lytic life cycle. The genome was found to contain 96 open reading frames (ORFs), as predicted by RAST. Of these, 47 ORFs (49%) were identified with predicted functions, while 49 ORFs encoded hypothetical proteins with unknown functions. The genome’s organization is based on gene transcription direction, which allows for three distinct functional clusters.

**Figure 5:**
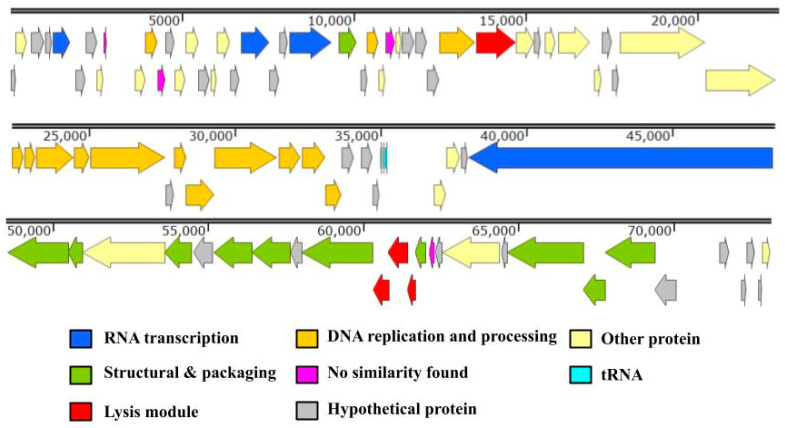
Schematic representation of the KPYAP-1 genome map. Genome organization of KYPYA-1. The coding DNA sequences (CDSs) are represented as arrows, color-coded to differentiate functional modules or regions. Green arrows indicate capsid and structural proteins and signify proteins related to morphogenesis; orange arrows denote CDSs involved in DNA or RNA metabolism, replication, and regulation; red arrows represent CDSs related to phage lysis; blue arrows indicate RNA polymerase; grey arrows denote hypothetical proteins; and yellow arrows are CDSs which can hit to known annotated genes but do not belong to phage-related genes. Additionally, the positions of tRNA genes are marked with light blue arrows. Areas with “No similarity” are marked in pink. This map was generated to visualize the genomic structure and highlight functional regions of the KYPYA-1 genome. The genome map was constructed by Snap Gene (https://www.snapgene.com/) (accessed on 15 August 2024).

The first cluster (ORF1–ORF64) includes genes involved in DNA or RNA metabolism, replication, and regulation, all transcribed in the right direction. The second cluster (ORF65–ORF86) contains genes related to virion structure, DNA packaging, and host lysis, transcribed in the opposite direction. The third segment consists of ten hypothetical ORFs (ORF87–ORF96) located at the 3′ end of the genome, transcribed in the right direction (see Figure 5). Most ORFs start translation at the AUG codon, with only three starting at the GUG codon (ORFs 20, 48, and 54). A detailed list of the proteins can be found in Table S1. ORF5 encodes the RNAP1 subunit A (similar to gp2 in the N4 phage), and ORF23 encodes the RNAP1 subunit B. Among the ORFs between ORF5 and ORF23, three are predicted to have specific functions: the peptidoglycan-binding domain protein (ORF13), the GTG-binding domain protein (ORF14), and ADP-ribosylglycohydrolase (ORF21). ORF26 (RNAP2) is followed by ORF27, which encodes a potential capsid-decorating protein with an Ig-like domain, typical for N4-like phages. The cluster of ORFs related to DNA synthesis generally follows the ORFs involved in RNA synthesis (see Figure 5, Table S1). Additionally, four tRNA genes for asparagine (GTT), proline (TGG), glutamine (TTG), and leucine (CAG) are grouped at the 3′ end of the first cluster. The second cluster includes ORFs for vRNAP (ORF65), structural proteins and phage capsid assembly (ORF66-74), release of mature phage particles (ORF75-77), and DNA packaging proteins, large (ORF85) and small (ORF86) terminase subunits. The lysis module features Rz/Rz1-like spanin (ORF75), SAR-endolysin with an N-acetylmuramidase domain (ORF76), and holin (ORF77) with two transmembrane helical domains of Class II [26]. ORF81 and ORF83 are two tail fiber proteins with no homologs in KP8. A list of homologs is available in Table S2.

### 2.6. Proteomic Analysis

**Figure 6:**
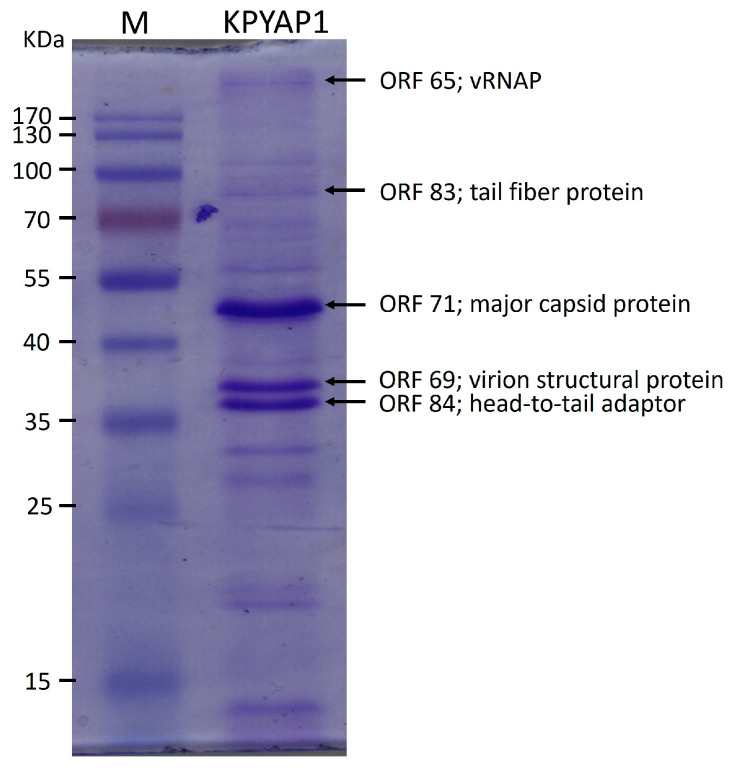
SDS-PAGE analysis of KPYAP-1 virion proteins. The lanes consist of M, which represents the PageRuler™ Prestained Protein Ladder (Thermo Fisher Scientific Inc., Waltham, MA, USA), and KPYAP-1, indicating the structural proteins of phage KPYAP-1. The molecular mass marker proteins are labeled on the left to show their relative migration, while proteins identified through tandem mass spectrometry (MS/MS) are marked on the right.

In addition, we performed a virion protein analysis using sodium dodecyl sulfate–polyacrylamide gel electrophoresis (SDS-PAGE). This analysis identified three major protein bands along with about ten smaller bands, covering a range of molecular weights from 15 to 180 kDa (Figure 6). Following this, we isolated five of these protein bands and analyzed them using tandem mass spectrometry (MS/MS). This analysis confirmed the presence of vRNAP and four structural proteins as annotated in the genome, including the predicted major coat protein, tail fiber, virion structural protein, and head-to-tail adaptor. However, some putative structural proteins were not identified through this method, likely due to their lower abundance.

### 2.7. Phylogenetic Analysis of KPYAP-1

**Figure 7:**
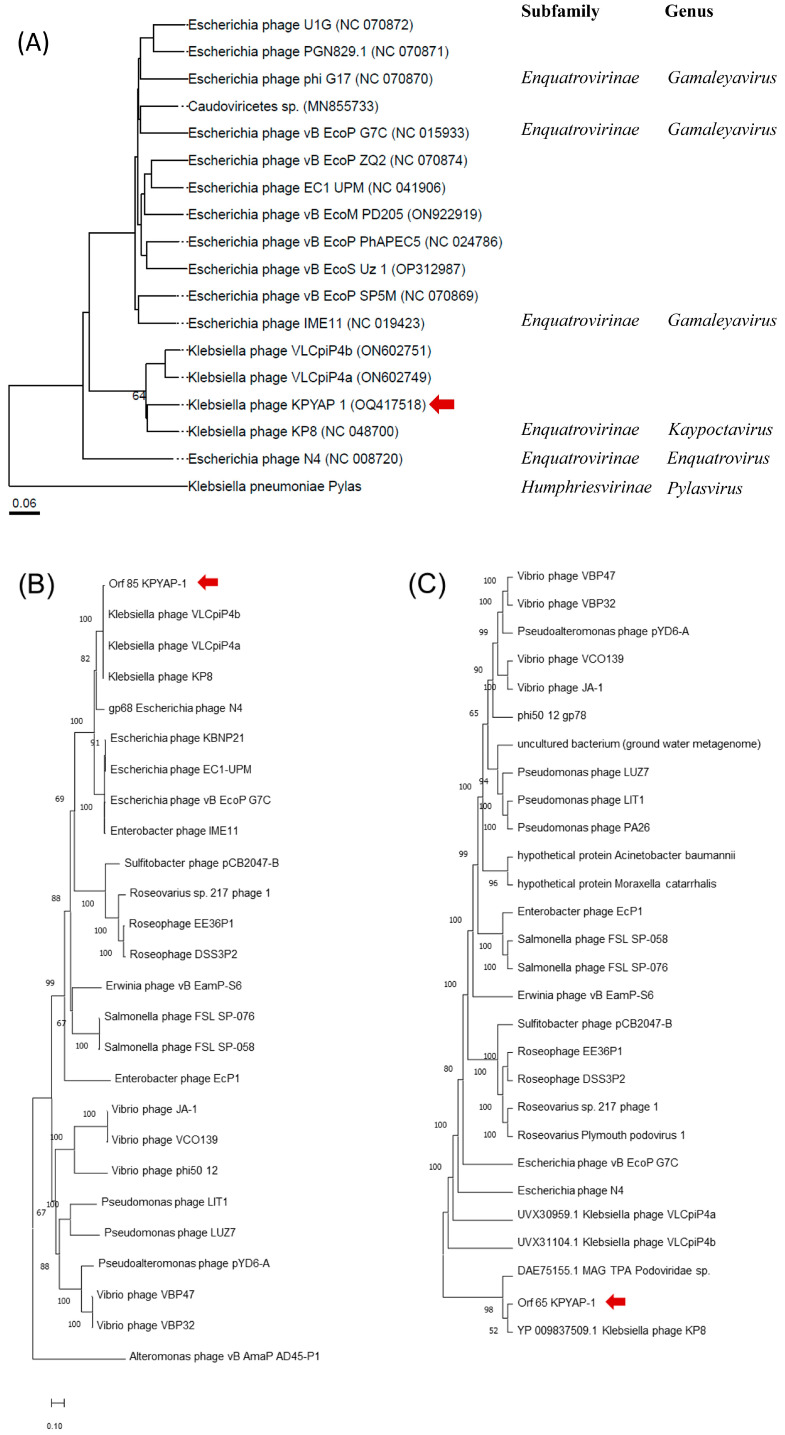
Taxonomic grouping and phylogenetic analysis of KPYAP-1. (**A**) Phylogenomic analysis was performed using a whole proteome approach with VICTOR. (**B**) The amino acid sequences of the large terminase subunits, and (**C**) Virion RNA polymerase was analyzed with Molecular Evolutionary Genetics Analysis Version 11 (MEGA11) software and showed well clustered with KP8. A phylogenetic tree was constructed using the neighbor-joining method with 1000 bootstrap replicates for reliability. The red arrows indicate the taxonomic position of KPYAP-1.

To determine the taxonomic identity of KPYAP-1, a comprehensive proteomic phylogenetic analysis was performed using VICTOR (https://ggdc.dsmz.de/victor.php) (accessed on 15 November 2022). The analysis placed KPYAP-1 in the same clade as KP8 and as closely related to the *Escherichia* phage N4, although in a different taxonomic group (Figure 7A). The virion RNA polymerase is unique and conserved in N4-like phages [27], while the large terminase is conserved, indicating the packaging mechanism [28]. To further explore the phylogenetic relationship of KPYAP-1 with other N4-like phages, a phylogenetic tree was constructed using MEGA11, based on selected large terminase subunits (Figure 7B) and the virion RNA polymerase (Figure 7C). The virion RNA polymerase of KPYAP-1 clustered with KP8 (Figure 7C). The large terminase subunit grouped KPYAP-1 with KP8 and two similar *Klebsiella* phages, VLCpiP4a and VLCpiP4b (Figure 7B). Therefore, we conclude that KPYAP-1 belongs to the *Schitoviridae* N4-like family and is the same as the K8 cluster within the *Enquatrovirinae* subfamily, and *Kaypoctavirus* genus.

### 2.8. Genome Comparisons of KPYAP-1

**Figure 8:**
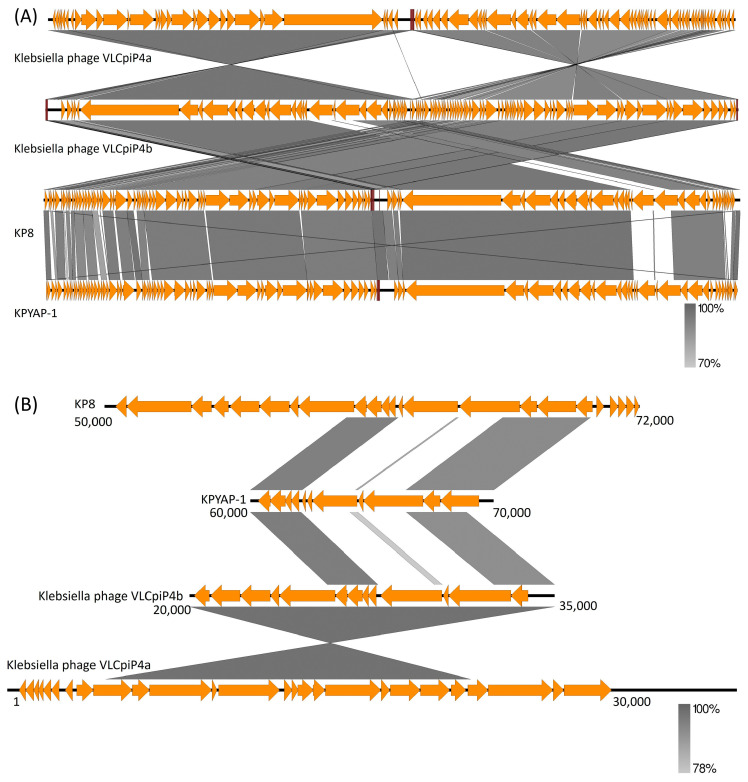
Genomic comparison among closely related genomes of KPYAP-1. (**A**) Comparative genomic analysis of four closely related phage genomes generated by Easyfig, showing colinear genomic organization. Continuous lines indicate sequence similarity, while gaps denote no detectable similarity. (**B**) Zooming into nucleotide positions 60,000 to 70,000 of the reference genome KYPYA-1.

Genome BLAST analysis identified two more closely related *Klebsiella* phages, VLCpiP4a and VLCpiP4b, in addition to KP8. The genomes of these closely related phages were compared with KPYAP-1 using Easyfig. There was a high similarity among these genomes, with a shared synteny in genetic organization (Figure 8A). Notably, an area around nucleotide positions 62,000 to 68,000 that includes two tail fiber proteins (ORF81 and ORF83) showed no similarity (Figure 8B). This difference was also evident when compared to the original *Escherichia coli* N4 phage using VIPTree analysis (Figure S1). A difference in the tail fiber protein was observed when comparing the tail fiber protein of KPYAP-1 (ORF83) and KP8 (YP_009837524.1) using BLASTp. The tail fiber proteins of KPYAP-1 and KP8 have amino acid lengths of 818 and 755, respectively. BLASTp results showed that the KPYAP-1 amino acid positions 259–326 have 33% identity with the KP8 amino acid positions 78–149. From this result, we can conclude that there is a significant difference in the tail fiber proteins between these two phages, which affects the host recognition, marking it as a novel phage.

### 2.9. Therapeutic Effect of KPYAP-1 on Kp20-Infected Zebrafish

Due to its genetic, anatomical, and physiological homology with mammals, the small tropical freshwater bony fish zebrafish (*Danio rerio*) has become a widely used model in the fields of behavioral, genetic, and infectious disease research [29]. Therefore, this study employed a Kp20-infected zebrafish model to assess the effectiveness of phage KPYAP-1 in the animal.

**Figure 9:**
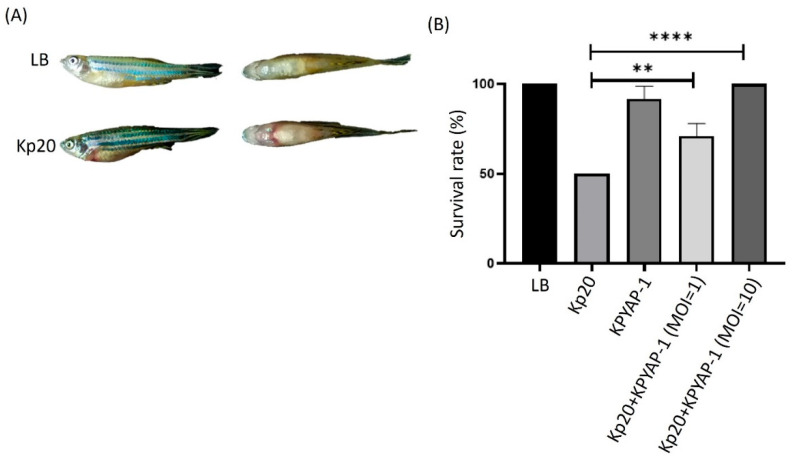
The pathogenicity of *K. pneumoniae* and the therapeutic effects of KPYAP-1 were assessed using a zebrafish model. (**A**) Zebrafish were injected with either Luria–Bertani (LB) broth medium or Kp20. The images show both side and top views of the zebrafish in the left and right panels, respectively. (**B**) Survival rates of the fish were compared between the LD50 group and different MOIs. Each experimental group included eight zebrafish, and the tests were conducted in triplicate. Significant differences are denoted by asterisks (** *p* ≤ 0.01; **** *p* ≤ 0.0001).

To determine the therapeutic effect and the safety of using KPYAP-1 for treatment, the fish were injected with Kp20 alone or with Kp20 and KPYAP-1 at varying MOIs, and examined after 24 h. All the fish survived (100%) in the control Luria–Bertani (LB) group and when injected with KPYAP-1 alone, which proves that it is safe to use in treatment. The survival was 50% in the group infected with the LD_50_ (1.8–3.5 × 10^7^ CFU/20 µL) of Kp20. Infected fish exhibited symptoms characterized by a swollen abdomen and bleeding (Figure 9A). The survival was 91.6% in the group infected with Kp20 and KPYAP-1 at an MOI of 10, whereas the survival was 70.6% in the group with Kp20 and KPYAP-1 at an MOI of 1. A statistically significant difference was observed in the survival rate of fish, regardless of whether the MOI was 1 or 10. Moreover, the survival rate increased with a higher MOI, indicating a positive correlation between MOI levels and fish survival (Figure 9B).

## 3. Discussion

Carbapenem-resistant *K. pneumoniae* poses a significant threat, drawing international attention because of its high mortality rate, which surpasses 40% among those infected [4]. In recent years, the K62 *K. pneumoniae* strain, a high-risk multidrug-resistant clonal lineage, has emerged as a prevalent serotype not only in Taiwan [10] but also in China [29] and Bangladesh [30]. In response to this threat, phage therapy emerges as a promising alternative to antibiotics, offering benefits such as swift bactericidal action, precise targeting, and low inherent toxicities [31]. Phages display high specificity towards their host bacteria, and bacterial strains can vary significantly between countries owing to diverse geographical distributions. In this study, a clinical bla*_OXA-48_*-producing CRKP with the polysaccharide capsule K62 serotype was used as the host. A lytic phage (KPYAP-1) was isolated from hospital sewage in Hualien, Taiwan, and identified as an N4-like phage. This particular phage strain is rarely documented among *K. pneumoniae* phages, especially regarding its specificity towards the K62 serotype. Biological characterization reveals that KPYAP-1 rapidly adsorbs to its host, achieving 99% adsorption within 10 min, regardless of the MOI. It effectively suppresses host bacterial growth within 8 h, leading to cell lysis with an average release of approximately 473 PFUs per infected cell. Interestingly, a halo zone is often observed around lysis plaques of N4-like phages, such as the closely related phage KP8 [32], and is believed to result from the action of phage-associated tail fibers with capsule-degrading enzyme activity [33]. However, no halo is present around the plaques produced by KPYAP-1. This suggests that, despite the annotation of two tail fiber proteins (ORF81 and ORF83) in the KPYAP-1 genome, they may not be expressed or active, leading to the distinct appearance of lysis plaques compared to other N4-like phages. Moreover, KPYAP-1 demonstrates enhanced activity and stability within a pH range of 3–11 when stored at 4 °C compared to 37 °C, which is potentially advantageous for clinical applications.

Whole genome analysis plays a crucial role in characterizing new phages and filling in the gaps left by in vitro studies. The genome of KPYAP-1 demonstrates gene synteny typical of N4-like phages, especially with KP8, and includes several homologs such as the large virion RNA polymerase (ORF65), RNA polymerase II (ORF26), and a single-stranded DNA-binding protein (ORF57), all of which are N4 homologs [34]. Additionally, the lysis cassette of KPYAP-1 shares similarities with that of N4, featuring a signal-anchor-release (SAR) endolysin with an N-acetylmuramidase domain [35], a holin, and an integrated inner/outer spanin pair [36]. The gene cluster predicted to encode spanin, endolysin, and holin suggests how KPYAP-1 may lyse infected bacterial cells to release newly formed phage particles at the end of the lytic cycle. Furthermore, phylogenetic tree analysis revealed that phage KPYAP-1 belongs to the *Enquatrovirinae* subfamily, *Kaypoctavirus* genus, and is most closely related to phage KP8 isolated in Russia [32], but less related to the other N4-like phage, Pylas, of *K. pneumoniae,* which was isolated in Texas, USA, (MH899585). They are separated into different genera within the *Enquatrovirinae* subfamily. Notably, KPYAP-1 exhibited a very narrow host range and only infected the K62 serotype, but the host strain of its closest homologous phage, KP8, is another unreported novel capsule type [32]. Given the notable distinctions observed in the tail fiber region (Figure 8), it is intriguing to note that ORF81 and ORF83 were identified as putative tail fiber proteins, a feature not commonly found in known N4-like phages [35] but rather in long-tailed phages. ORF81, as identified by InterProScan, showed the highest similarity (91% identity over 91% coverage) to the tail fiber protein of *Klebsiella* phage Kpn BU9 (OR145793.1), which belongs to the *Caudoviricetes* class and is characterized by a long noncontractile tail. Conversely, ORF83 exhibits the highest similarity (57% identity over 84% coverage) to the tailspike protein of *Klebsiella* myovirus vB_KqM-Westerburg (LR881137.1). Therefore, we speculate that host recognition may be influenced by this region.

Furthermore, therapeutic phages may carry undesirable characteristics, such as virulence and resistance genes, highlighting the critical role of whole genome sequencing in detecting these traits [37]. In silico analysis of the phage revealed no genes associated with lysogeny, antibiotic resistance, or bacterial virulence. Thus, the genomic analysis of KPYAP-1 strongly indicates that this phage is both safe and suitable for use as a therapeutic agent. A new generation of phage-derived lysins also has been proposed as a solution to overcome the existing challenges of phage therapy, making this treatment more accessible [38]. Consequently, the lytic module of phage KPYAP-1, which includes endolysin, holin, and spanin, holds significant potential for advancing medical applications. For instance, optimizing the lysis clock mechanism in holin–endolysin-engineered bacterial cells could enhance thermostability, increase solubility for better bioavailability, offer either highly targeted or broad-spectrum activity depending on the infection, provide protease resistance for gut applications, and be cost-effective to produce.

## 4. Materials and Methods

### 4.1. Bacterial Strains

Bacterial strains were isolated by standard culturing of clinical samples referring to the Department of Medical Research, Hualien Tzu Chi Hospital, Buddhist Tzu Chi Medical Foundation, Hualien, Taiwan. The isolated microorganisms were confirmed by colony PCR, which was conducted with the forward primer (5′-ATTTGAAGAGGTTGCAAACGAT-3′) and reverse primer (5′-TTCACTCTGAAGTTTTCTTGTGTTC-3′) for amplification of 16S rDNA followed by Sanger sequencing. The stocks of bacterial strains were stored in 25% glycerol at −80 °C for further experiments. Running cultures were picked from a single colony from the LB (Becton, Dickinson and Company, Franklin Lakes, NJ, USA) agar plate and inoculated in LB broth at 37 °C in a shaking incubator at 150 rpm for further growth. The growth of cells was observed by measuring optical density at 600 nm (OD_600_), with an OD_600_ value of 1 approximately corresponding to 3 × 10^8^ CFU/mL.

To determine *K. pneumoniae* capsular types (K-types), PCR amplification of a 580 bp fragment of the wzi gene was achieved using the primers wzi_for2 (GTG CCG CGA GCG CTT TCT ATC TTG GTA TTC C) and wzi_rev (GAG AGC CAC TGG TTC CAG AA[C or T] TT[C or G] ACC GC) [9]. O genotypes were performed by PCR, using a method described by Fang et al. [39], except that we did not differentiate O2ac from O2a. Multilocus sequence typing (MLST) was analyzed with seven genes (*gapA*, *infB*, *mdh*, *pgi*, *phoE*, *rpoB*, and *tonB*) according to the protocol described on the *K. pneumoniae* MLST website (www.pasteur.fr/mlst (accessed on 15 August 2024)) [40]. The MLST database was used to assign alleles and sequence types (STs).

### 4.2. Screening for Carbapenemase Production

Bacterial isolates with an inhibition zone of ≤19 mm against imipenem and meropenem were selected to screen isolates that produce carbapenemase, and further confirmed with a CHROMagar^TM^ mSuperCARBATM (CHROMagar, Paris, France) plate. If the colony showed a metallic blue color, it was determined to be a carbapenemase producer.

### 4.3. Detecting the bla_OXA-48_ Gene

For gene amplification, colony PCR was assessed with the OXA-48F (5′-TTGGTGGCATCGATTATCGG-3′) and OXA-48R (3′-GAGCACTTCTTTTGTGATGGC-5′) as forward and reverse primers [41]. The thermal cycling process consisted of initial denaturation at 94 °C for 10 min, with 30 cycles of denaturation at 94 °C for 40 s, annealing at 57 °C for 40 s, and extension at 72 °C for 50 s, with a final extension at 72 °C for 7 min. The amplicons were subjected to gel electrophoresis, and the amplified products were 743 bps in size.

### 4.4. Phage Isolation and Purification

The steps of phage isolation and purification were followed according to our previous study [42]. Briefly, 100 mL of unsterilized sewage samples were collected from around Buddhist Tzu Chi General Hospital, Hualien, Taiwan and centrifuged at 3000× *g* for 20 min. The supernatant was taken and filtered through a sterile 0.45 μm filter to remove the bacteria from the sewage samples. To enrich the phage population, the filtered supernatant was incubated overnight with Kp20, a clinically isolated CRKP strain, during its logarithmic growth phase (OD_600_ = 0.5–0.7) at 37 °C and 200 rpm/min. Subsequently, the mixture was centrifuged at 2000× *g* for 20 min at 4 °C. The resulting supernatant was filtered through a sterile 0.22 μm filter to eliminate bacteria. Subsequently, 100 μL of the supernatant and 100 μL of the corresponding bacterial culture were combined in 4 mL of 0.7% agar LB medium and spread on the surface of a 1.5% agar LB medium plate, followed by overnight incubation at 37 °C. Clear phage plaques were identified the next day and purified using a sterile toothpick. Each phage plaque was repeatedly isolated until a consistent and stable morphology was observed.

Isopycnic centrifugation was employed via CsCl gradient purification for further purification. A high-titer phage lysate (10^12^ PFU) was first precipitated by centrifugation at 15,000× *g* for 2 h at 4 °C using an Avanti JXN-26 centrifuge with a JA-25.50 rotor (Beckman Coulter, CA, USA). The resulting phage pellet was resuspended in SM buffer (0.05 M Tris-HCl, pH 7.5, containing 0.1 M sodium chloride, 0.008 M MgSO_4_·7H_2_O, and 0.01% gelatin). This suspension was layered onto a CsCl block gradient with densities ranging from 1.7 to 1.3 g/mL and then centrifuged at 25,000 rpm for 3 h at 4 °C using an SW 41 Ti rotor in an Optima XPN-100 ultracentrifuge (Beckman Coulter). The banded phage particles were carefully collected, dialyzed in SM buffer at 4 °C for 24 h, and subsequently stored at 4 °C for future applications.

### 4.5. Phage Characterization

#### 4.5.1. Morphological Observation of Phage by TEM

To assess phage morphology, a negatively stained sample was prepared for examination under TEM. Approximately 10 μL of dialyzed phage solution (approximately 10^10^ PFU) was deposited onto a formvar-coated copper grid (300 mesh) and then stained with 2% uranyl acetate. The grid was allowed to dry before being observed under a Hitachi H-7500 TEM instrument located in Tokyo, Japan. Morphological characteristics were examined using an acceleration voltage of 80 kV, and images were captured with a CCD camera for further analysis.

#### 4.5.2. Host Range Analysis of Phage

The host range specificity of the phage was determined based on whether the phage formed a clear spot on the 47 CRKP clinical isolates. Briefly, 5 μL of phage suspension (10^8^ PFU/mL) was spotted onto bacterial overlay LB agar plates. After overnight incubation at 37 °C, the plates were analyzed for the clear zone on the spotted area to confirm phage infectivity.

#### 4.5.3. One-Step Growth and Adsorption Efficiency of Phage

To generate the one-step growth curve, we followed a modified version of a previously reported method [42]. Initially, a culture of the host bacteria (5 mL) was grown until the OD_600_ reached 0.6 to 0.8. Subsequently, bacterial cells (1 mL, approximately 1.0 × 10^8^ CFU) were pelleted via centrifugation (8000× *g* for 5 min at room temperature), resuspended in 0.9 mL of SM buffer, and mixed with 0.1 mL of a phage suspension (1.0 × 10^5^ PFU/mL). The mixture was then incubated on ice for 30 min. After centrifugation (8000× *g* for 2 min at room temperature), the supernatant containing free phage was removed, and the pellet was suspended in 15 mL of LB medium. The mixture was incubated at 37 °C with shaking at 200 rpm. Samples were collected at 5 min intervals up to 35 min, and the phage titer was determined using the double-layer agar technique. The burst size was calculated as the ratio of the final count of liberated phage particles to the initial count of infected bacterial cells during the latent period [43].

For phage adsorption analysis, we conducted it as previously described [42]. Briefly, bacterial cells were infected with the phage at an MOI of 0.001 and incubated at 37 °C with shaking. Samples (100 µL) were collected after 0, 2, 4, 6, 8, and 10 min. The aliquots were then centrifuged at 12,000 rpm for 5 min, and a double-layer agar experiment was performed with the supernatants to determine the number of unadsorbed phages present. The phage adsorption efficiency (%) was calculated using the following equation: (initial titer of phage—titer of unadsorbed phages present in the supernatant)/initial titer of phage, multiplied by 100.

#### 4.5.4. Time-Dependent Bacteriolytic Effect of Phage

An in vitro microtiter plate assay was conducted with slight modifications to assess the time-dependent bacteriolytic effect of the phage [44]. Initially, the host bacterial inoculum (~10^8^ CFU/mL) was prepared by diluting the overnight culture in LB medium and adjusting it to an OD_600_ of ~0.3. Phage lysates were titrated and adjusted to concentrations of 10^5^, 10^6^, 10^7^, 10^8^, and 10^9^ PFU/mL using SM buffer. For each assay, 180 µL of the adjusted bacterial inoculum in LB medium was mixed with 20 µL of phage lysates with varying doses in sterile, untreated Falcon^®^ 96-well transparent plates (Thermo Fisher Scientific Inc.) to achieve final MOI values of 0.001, 0.01, 0.1, 1, and 10, respectively. These plates were then incubated at 37 °C, and the growth of bacteria was monitored by measuring the OD_600_ at 1 h intervals for 12 h using a microtiter plate reader (Clariostar Plus, BMG Labtech, Offenburg, Germany), including the initial measurement at time 0. Growth curves were generated by plotting the OD values after baseline adjustment against time. Each assay was conducted with three biological replicates to ensure the reliability and reproducibility of the results.

#### 4.5.5. Phage Stability at Varying Temperatures and pH Values

The stability of phage lysate was determined by phage titer determination after incubation for 1 h at a pH range of 3–11 and varying temperatures (4, 37, 50, and 65 °C). Briefly, the phage lysate was diluted with LB (at the volume proportion 1:9) and incubated under the conditions described. The mixture was then withdrawn shortly, and serial 10-fold dilutions were used for double-layer agar plating. Phages without any treatment were the control. After overnight incubation at 37 °C, the percentage of remaining plaque-forming phages was calculated.

#### 4.5.6. Phage DNA Isolation and Determination of Genome Size and Restriction Patterns by PFGE

Phage DNA was extracted following previously described methods with some modifications based on an earlier study [42]. Briefly, the phage suspension was treated with 1 µg/mL DNase I and 10 µg/mL RNase A (Promega, Madison, WI, USA) for 3 h. Subsequently, phage particles were concentrated using 20% PEG 6000 and 2.5 M NaCl, followed by centrifugation at 18,000 rpm (Beckman Coulter Avanti JXN-26 centrifuge, JA-25.50 rotor; Beckman Coulter, Brea, CA, USA) for 2 h at 4 °C. The concentrated phage was then subjected to phenol/chloroform extraction, and the phage DNA was precipitated using ethanol.

The genome size was determined using a CHEF-DR III System (Bio-Rad Laboratories, Hercules, CA, USA) at 6 V/cm with pulse ramps from 5 to 20 s for 10 h at 14 °C in 0.5× Tris-borate-EDTA (TBE) buffer. The size was calculated by comparing the results to a standard concatenated λ PFG ladder ranging from 48.5 to 1018 kb (New England Biolabs, Ipswich, MA, USA).

For restriction pattern analysis, the phage DNA was digested with restriction enzymes following the conditions given by the manufacturer (Fermentas, Waltham, MA, USA). The digested DNA samples were analyzed in PFGE with the conditions mentioned above. The λ DNA-HindIII Digest (New England Biolabs) marker was used to compare the size of the digested fragments.

#### 4.5.7. Phage Genome Sequencing and Characterization

The phage DNA genome was sequenced by AllBio. Science. Inc (Taichung, Taiwan). The isolated phage DNA (~5 μg) was made into fragments of 500 bps using the Covaris ultrasonic crusher (Covaris, LLC., Woburn, MA, USA). The nucleotide base A was added to the 3′ end in order to execute end repair prior to sticky end formation. After the DNA segments flanked by adapters were amplified by PCR, electroporation was used to extract the target fragment. Using Bioanalyzer (Agilent, Santa Clara, CA, USA), the PCR products were cleaned and validated. A paired-end 150 sequencing of the qualifying libraries was performed using the HiseqXten/Novaseq/MGI2000 system (Illumina, San Diego, CA, USA). The genome annotation was done by Rapid Annotations Subsystems Technology (RAST) [45,46,47] and was manually annotated by BLASTp [48]. The phage terminal repeats were found by Phageterm, which is available on the Galaxy-based server [49] and confirmed by RepEx [50]. A phage genome organization map was constructed using Snap Gene v6.1.2 (GSL Biotech; available at https://www.snapgene.com/; accessed on 9 September 2022) [51]. The tRNAs in the genome were determined by ARAGORN v1.2.41 [52]. The phylogenetic analysis and the evolutionary history were inferred using the Neighbor-Joining method in MEGA11 [53,54]. The whole genome comparison with the closely related phages was done by VICTOR (https://ggdc.dsmz.de/victor.php) (accessed on 15 November 2022) and the pairwise comparisons of the closely related phage sequences were conducted by the Genome BLAST Distance Phylogeny (GBDP) method [55,56]. The OPTSIL program was used to determine the taxon boundaries at the species, genus, and family levels, using the recommended clustering thresholds, an F value (fraction of links required for cluster fusion) of 0.5 [55,57,58], and Easyfig [59]. The presence of antibiotic resistance genes was found by ResFinder 4.1 [48,60,61], and the presence of virulence genes was examined by virulence finder 2.0 (https://cge.cbs.dtu.dk/services/VirulenceFinder/) (accessed on 13 September 2022).

Phage KPYAP-1’s annotated whole genome was uploaded to the GenBank database with the accession number OQ417518.

#### 4.5.8. Phage Structural Protein Analysis and Mass Spectrometry

Phage particles were purified from phage preparation by CsCl gradient ultracentrifugation. Proteins from the purified phage KPYAP-1 particles were separated using Tris-glycine SDS 12% (*w*/*v*) polyacrylamide gel electrophoresis and visualized by Coomassie R250. Gel fragments containing individual protein bands were cut out from the gel, and trypsin digestion was conducted as described previously [62]. Briefly, the desired proteins from the stained gel were subjected to in-gel digestion. The extracted fragments of the gel were reduced and subsequently alkylated. The peptides were obtained from the gel fragments after trypsin digestion (Promega, Madison, WI, USA). The UltiMate 3000 RSLCnano system coupled to a Q Exactive mass spectrometer (Thermo Fisher Scientific Inc.) was used to analyze the peptides. The results of the mass spectrometer were compared against the peptide profile predicted from the KPYAP-1 genome sequence.

#### 4.5.9. Evaluating Phage Effectiveness against *K. pneumoniae*-Infected Zebrafish

Zebrafish (*Danio rerio*) were utilized in this study and were raised following standard protocols at the fish core facility of Tzu Chi University. The fish were housed in 9 L tanks with a 14 h light and 10 h dark cycle at 28 °C. Both male and female zebrafish were included in the study. All experimental procedures were conducted in accordance with the regulations and guidelines of the Tzu Chi University Institutional Animal Care and Use Committee (IACUC) (approval number 111091).

The experimental procedures were performed as previously described [63]. Prior to phage treatment, the lethal dose (LD_50_) of *K. pneumoniae* Kp20 for zebrafish was determined. Adult, disease-free zebrafish with a size range of 3–3.5 cm were injected with various doses of Kp20 (3 × 10^6^, 3 × 10^7^, and 3 × 10^8^ CFU) via the cloaca using an insulin needle. Each fish in the experimental group (*n* = 8) received an injection of 20 μL of purified phage (3 × 10^9^ PFU), while the control group received an injection of 20 μL of SM buffer for phage safety testing.

To evaluate the efficacy of phage treatment against *K. pneumoniae* infection in zebrafish, the phage was administered into the zebrafish cloaca 30 min after injection with *K. pneumoniae*. Prior to injection, the fish were anesthetized with 0.2% tricaine. Following injection, the fish were transferred to separate water tanks, and their survival rate was monitored at room temperature by observing them every 6 h for 24 h. Any behavioral changes (such as jumping from the tank, slow or rapid movement, gulping for air, or staying at the bottom of the tank) were recorded and considered as potential signs of morbidity.

### 4.6. Statistical Analysis

All experiments were performed in triplicate and the results are presented as mean  ±  standard deviation (SD). Graphs and statistical analyses were generated using GraphPad Prism 9.1.1 software. Statistical significance was determined using both the two-tailed Student’s *t*-test and ANOVA, with a threshold of *p*  <  0.05.

## 5. Conclusions

As the spread of MDR *K. pneumoniae* persists, utilizing phage therapy emerges as a hopeful substitute for antibiotics in combating this superbug. We isolated a lytic phage, KPYAP-1, from hospital sewage, specifically targeting carbapenem-resistant *K. pneumoniae* and the K62 capsule serotype. Phage KPYAP-1 is noted for its strong environmental adaptability, precise lytic specificity, short incubation period, and an adequate burst size. Animal studies demonstrated that KPYAP-1 significantly improved survival rates, indicating its potential for clinical use. Further, in vivo studies and clinical practices are required to confirm the efficacy of KPYAP-1 in treating severe CRKP infections.

## Data Availability

The data presented in this study are available on request from the corresponding author.

## Supplementary Materials

The following supporting information can be downloaded at https://www.mdpi.com/article/10.3390/ijms25179595/s1.
