# Supplementary material for: In Vitro and In Vivo Assessments of Newly Isolated N4-like Bacteriophage against ST45 K62 Capsular-Type Carbapenem-Resistant Klebsiella pneumoniae: vB_kpnP_KPYAP-1
Source: Int J Mol Sci. 2024 Sep 4;25(17):9595. doi: 10.3390/ijms25179595 (PMC11395603; doi:10.3390/ijms25179595)
Supplement: Supplementary file 1 [file ijms-25-09595-s001.zip › Table S1 and S2-v2.pdf]

Table S1. Annotated genome of KPYAP-1

ORF	ORF position bp	Amino acid length	Strand	% Identity with GenBank phage protein sequences by BlastP	Predicted function	pI/Mw (kDa)
ORF 1	1-150	49	+	97.67% (Podoviridae sp. [DAG71669.1])	hypothetical protein	4.20 / 5.10
ORF 2	147-470	107	+	99.07% (Podoviridae sp. [DAE75218.1])	hypothetical protein	5.16 / 12.02
ORF 3	584-982	132	+	93.18% (Podoviridae sp. [DAE75219.1])	hypothetical protein	5.17 / 14.82
ORF 4	993-1205	70	+	71.43% (Klebsiella phage KP8 [YP_009837444.1])	hypothetical protein	5.21 / 7.90
ORF 5	1239-1733	164	+	95.73% (Klebsiella phage KP8 [YP_009837446.1])	RNA polymerase 1 subunit A	5.07 / 18.18
ORF 6	1878-2180	100	+	82% (Klebsiella phage VLCpiP4a [UVX31016.1])	hypothetical protein	9.86 / 11.87
ORF 7	2177-2515	112	+	96.43% (Podoviridae sp. [DAG71679.1])	hypothetical protein	5.82 / 13.17
ORF 8	2512-2703	63	+	96.83% (Klebsiella phage KP8 [YP_009837449.1])	putative helical domain containing protein	9.52 / 6.602
ORF 9	2703-2798	31	+	No significant similarity found		3.57 / 3.59
ORF 10	2926-3123	65	+	95.24% (Klebsiella phage VLCpiP4b [UVX31145.1])	hypothetical protein	10.61 / 7.20
ORF 11	3124-3342	72	+	54.24% (Serratia liquefaciens [WP_261278877.1])	hypothetical protein	6.82 / 8.042
ORF 12	3405-3620	71	+	84.51% (Klebsiella phage KP8 [YP_009837453.1])	hypothetical protein	5.29 / 8.07
ORF 13	3617-3925	102	+	93.14% (Klebsiella phage KP8 [YP_009837454.1])	putative peptidoglycan binding domain containing protein	6.09 / 11.56

ORF	ORF position bp	Amino acid length	Strand	% Identity with GenBank phage protein sequences by BlastP	Predicted function	pI/Mw (kDa)
ORF 14	3922-4278	118	+	90.68% (Klebsiella phage KP8 [YP_009837455.1])	putative DNA processing chain A	9.10 / 13.26
ORF 15	4280-4504	74	+	No significant similarity found		6.00 / 8.13
ORF 16	4501-4779	92	+	94.57% (Klebsiella phage VLCpiP4b [UVX31148.1])	hypothetical protein	9.93 / 10.82
ORF 17	4776-5096	106	+	98.06% (Klebsiella phage KP8 [YP_009837457.1])	antirepressor protein	4.97 / 12.03
ORF 18	5093-5473	126	+	89.68% (Klebsiella phage KP8 [YP_009837458.1])	DUF4326 domain-containing protein	6.95 / 13.95
ORF 19	5466-5810	114	+	91.23% (Klebsiella phage VLCpiP4b [UVX31150.1])	hypothetical protein	5.94 / 13.15
ORF 20	5815-6012	65	+	93.85% (Podoviridae sp. [DAE75238.1])	KaiB domain	9.24 / 7.64
ORF 21	6009-6389	126	+	95.24% (Klebsiella phage KP8 [YP_009837460.1])	ADP-ribosylglycohydrolase	6.63 / 13.82
ORF 22	6389-6661	90	+	70% (Shigella virus Moo19 [UEN68820.1])	hypothetical protein	9.55 / 10.47
ORF 23	6712-7527	271	+	98.89% (Podoviridae sp. [DAE75145.1])	DNA directed RNA polymerase	5.95 / 31.90
ORF 24	7520-7816	98	+	94.9% (Klebsiella phage VLCpiP4a [UVX31001.1])	hypothetical protein	4.94 / 10.85
ORF 25	7827-8078	83	+	93.98% (Klebsiella phage VLCpiP4b [UVX31156.1])	hypothetical protein	4.61 / 9.05
ORF 26	8125-9339	404	+	97.77% (Klebsiella phage KP8 [YP_009837466.1])	RNA polymerase 2 subunit A	5.29 / 45.92
ORF 27	9565-10080	171	+	97.66% (Klebsiella phage KP8 [YP_009837467.1])	capsid decorating protein	4.47 / 18.08

ORF	ORF position bp	Amino acid length	Strand	% Identity with GenBank phage protein sequences by BlastP	Predicted function	pI/Mw (kDa)
ORF 28	10190-10390	66	+	96.97% (Klebsiella phage VLCpiP4a [UVX30997.1])	hypothetical protein	10.46 / 7.03
ORF 29	10368-10718	116	+	99.04% (Klebsiella phage KP8 [YP_009837470.1])	HNH endonuclease	9.91 / 13.22
ORF 30	10715-10921	68	+	73.13% (Klebsiella phage KP8 [YP_009837471.1])	putative transmembrane helical domain containing protein	7.85 / 7.33
ORF 31	10914-11201	95	+	No significant similarity found		5.38 / 10.38
ORF 32	11211-11396	61	+	95.08% (Klebsiella phage KP8 [YP_009837472.1])	putative 2 transmembrane helical domains containing protein	9.66 / 7.19
ORF 33	11397-11756	119	+	92.44% (Podoviridae sp. [DAE75161.1])	hypothetical protein	6.57 / 13.31
ORF 34	11782-12135	117	+	97.44% (Klebsiella phage KP8 [YP_009837474.1])	hypothetical protein	6.04 / 13.20
ORF 35	12135-12482	115	+	93.91% (Klebsiella phage VLCpiP4a [UVX30991.1])	hypothetical protein	4.17 / 13.06
ORF 36	12493-13545	350	+	99.43% (Klebsiella phage KP8 [YP_009837476.1])	ATPase	5.61 / 39.45
ORF 37	13556-14707	383	+	100% (Klebsiella phage KP8 [YP_009837477.1])	metallopeptidase	5.18 / 43.82
ORF 38	14715-15242	175	+	98.86% (Klebsiella phage KP8 [YP_009837478.1])	dCTP deaminase	9.68 / 19.47
ORF 39	15245-15445	66	+	98.48% (Podoviridae sp. [DAG71622.1])	hypothetical protein	6.53 / 6.96
ORF 40	15561-15869	102	+	80.58% (Podoviridae sp. [DAG71623.1])	Shikimate kinase	9.99 / 11.88
ORF 41	15950-16888	312	+	97.12% (Klebsiella phage VLCpiP4b [UVX31172.1])	thymidylate synthase	6.38 / 35.68

ORF	ORF position bp	Amino acid length	Strand	% Identity with GenBank phage protein sequences by BlastP	Predicted function	pI/Mw (kDa)
ORF 42	16989-17225	78	+	97.44% (Klebsiella phage KP8 [YP_009837482.1])	hypothetical protein	4.70 / 8.60
ORF 43	17218-17526	102	+	99.02% (Klebsiella phage VLCpiP4b [UVX31174.1])	hypothetical protein	4.20 / 11.04
ORF 44	17519-17716	65	+	50.75% (Klebsiella phage VLCpiP4a [UVX30982.1])	hypothetical protein	8.96 / 7.92
ORF 45	17735-20254	839	+	93.68% (Klebsiella phage VLCpiP4b [UVX31176.1])	rIIA lysis inhibitor	9.15 / 94.82
ORF 46	20251-22287	678	+	98.53% (Klebsiella phage KP8 [YP_009837486.1])	rIIB-like protein	7.11 / 73.52
ORF 47	22347-22742	131	+	96.95% (Podoviridae sp. [DAG71628.1])	helix-turn-helix domain protein	6.97 / 14.84
ORF 48	22771-23127	118	+	90.68% (Klebsiella phage KP8 [YP_009837488.1])	triphosphate pyrophosphohydrolase	4.28 / 13.12
ORF 49	23164-24471	435	+	99.54% (Klebsiella phage KP8 [YP_009837489.1])	DNA helicase	6.29 / 49.08
ORF 50	24480-25010	176	+	97.73% (Podoviridae sp. [DAG71629.1])	Nucleotide modification associated domain 5	5.84 / 20.53
ORF 51	25019-27601	860	+	99.42% (Klebsiella phage KP8 [YP_009837491.1])	DNA polymerase I	5.34 / 97.60
ORF 52	27598-27897	99	+	100% (Podoviridae sp. [DAE75174.1])	hypothetical protein	4.68 / 10.89
ORF 53	27901-28326	141	+	87.23% (Podoviridae sp. [DAE75150.1])	TPA: nucelotide kinase	5.46 / 15.95
ORF 54	28313-29284	323	+	99.38% (Klebsiella phage KP8 [YP_009837495.1])	nuclease superfamily protein	6.81 / 37.30
ORF 55	29284-31431	715	+	100% (Klebsiella phage VLCpiP4b [UVX31186.1])	DNA primase	5.76 / 81.81

ORF	ORF position bp	Amino acid length	Strand	% Identity with GenBank phage protein sequences by BlastP	Predicted function	pI/Mw (kDa)
ORF 56	31489-32247	252	+	100% (Klebsiella phage KP8 [YP_009837497.1])	ATPase	5.12 / 28.68
ORF 57	32290-33084	264	+	100% (Klebsiella phage VLCpiP4a [UVX30968.1])	single strand DNA binding protein	5.41 / 28.42
ORF 58	33084-33641	185	+	98.92% (Klebsiella phage KP8 [YP_009837499.1])	holliday junction resolvase	8.32 / 20.23
ORF 59	33646-34083	145	+	93.1% (Podoviridae sp. [DAE75176.1])	hypothetical protein	4.40 / 15.94
ORF 60	34321-34725	134	+	94.78% (Klebsiella phage VLCpiP4a [UVX30965.1])	hypothetical protein	5.25 / 15.72
ORF 61	34715-34960	81	+	96.3% (Klebsiella phage KP8 [YP_009837503.1])	hypothetical protein	7.76 / 9.15
ORF 62	36814-37239	141	+	85.11% (Klebsiella phage VLCpiP4a [UVX30963.1])	hypothetical protein	4.58 / 15.40
ORF 63	37239-37733	164	+	98.78% (Klebsiella phage VLCpiP4a [UVX30962.1])	hypothetical protein	4.95 / 18.69
ORF 64	37743-37991	82	+	91.46% (Klebsiella phage VLCpiP4b [UVX31101.1])	hypothetical protein	6.72 / 9.23
ORF 65	48434-38022	347	-	98.65% (Klebsiella phage KP8 [YP_009837509.1])	virion RNA polymerase	5.44 / 378.46
ORF 66	50485-48536	649	-	98.92% (Klebsiella phage VLCpiP4b [UVX31105.1])	virion structural protein	5.48 / 69.95
ORF 67	50935-50495	146	-	97.26% (Klebsiella phage KP8 [YP_009837511.1])	structural protein	9.10 / 15.47
ORF 68	53607-50947	886	-	97.74% (Klebsiella phage KP8 [YP_009837512.1])	putative 4 transmembrane helical domains containing protein	4.66 / 97.56

ORF	ORF position bp	Amino acid length	Strand	% Identity with GenBank phage protein sequences by BlastP	Predicted function	pI/Mw (kDa)
ORF 69	54441-53611	276	-	98.19% (Klebsiella phage VLCpiP4b [UVX31108.1])	virion structural protein	4.78 / 30.27
ORF 70	55135-54518	205	-	98.54% (Podoviridae sp. [DAE75196.1])	hypothetical protein	6.20 / 23.19
ORF 71	56394-55189	401	-	98.5% (Podoviridae sp. [DAE75138.1])	major capsid protein	5.42 / 44.13
ORF 72	57636-56407	409	-	98.78% (Klebsiella phage KP8 [YP_009837516.1])	tape measure protein	4.42 / 44.82
ORF 73	58004-57654	116	-	98.28% (Klebsiella phage KP8 [YP_009837517.1])	hypothetical protein	4.68 / 13.23
ORF 74	60303-58018	761	-	98.95% (Klebsiella phage KP8 [YP_009837518.1])	portal protein	4.89 / 85.10
ORF 75	60812-60312	166	-	98.79% (Klebsiella phage KP8 [YP_009837519.1])	Rz/RzI spanin protein	8.79 / 18.89
ORF 76	61431-60793	212	-	98.39% (Klebsiella phage VLCpiP4a [UVX30948.1])	endolysin	9.27 / 22.85
ORF 77	61681-61415	88	-	98.86% (Klebsiella phage VLCpiP4a [UVX30947.1])	holin protein	9.51 / 10.23
ORF 78	61998-61666	110	-	81.25% (Klebsiella pneumoniae [WP_148831845.1])	hypothetical protein	5.52 / 12.29
ORF 79	62273-62130	47	-	No significant similarity found		3.98 / 5.34
ORF 80	62526-62332	64	-	81.25% (Klebsiella pneumoniae [WP_148831845.1])	hypothetical protein	4.18 / 6.90
ORF 81	64382-62541	613	-	92.8% (Klebsiella pneumoniae [MBD7746981.1])	right-handed parallel beta-helix repeat-containing protein	6.05 / 65.57

ORF	ORF position bp	Amino acid length	Strand	% Identity with GenBank phage protein sequences by BlastP	Predicted function	pI/Mw (kDa)
ORF 82	64628-64446	60	-	43.1% (Klebsiella phage vB_KqM-Westerburg [CAD5240872.1])	hypothetical protein	3.91 / 6.76
ORF 83	67093-64637	818	-	57.35% (Klebsiella phage vB_KqM-Westerburg [CAD5240870.1])	putative tail fiber protein	4.94 / 87.71
ORF 84	67797-67090	235	-	98.72% (Klebsiella phage KP8 [YP_009837526.1])	tail protein	4.97 / 27.08
ORF 85	69396-67804	530	-	99.06% (Klebsiella phage KP8 [YP_009837527.1])	large terminase subunit	5.21 / 60.60
ORF 86	70081-69389	230	-	100% (Klebsiella phage VLCpiP4b [UVX31123.1])	hypothetical protein	5.44 / 25.60
ORF 87	70255-70551	98	+	96.94% (Podoviridae sp. [DAE75207.1])	hypothetical protein	5.26 / 10.86
ORF 88	70808-71140	110	+	97.27% (Klebsiella phage VLCpiP4a [UVX30938.1])	hypothetical protein	7.83 / 12.58
ORF 89	71149-71487	112	+	88.99% (Podoviridae sp. [DAE75210.1])	hypothetical protein	5.25 / 13.18
ORF 90	71484-71810	108	+	83.33% (Klebsiella phage KP8 [YP_009837532.1])	hypothetical protein	7.92 / 11.99
ORF 91	71814-72005	63	+	54.1% (Klebsiella phage VLCpiP4a [UVX30936.1])	hypothetical protein	4.79 / 7.42
ORF 92	72025-72177	50	+	42% (Podoviridae sp. [DAG71664.1])	hypothetical protein	4.12 / 5.82
ORF 93	72170-72364	64	+	80.33% (Klebsiella phage VLCpiP4a [UVX30935.1])	hypothetical protein	6.16 / 7.26
ORF 94	72361-72633	90	+	94.44% (Klebsiella phage KP8 [YP_009837538.1])	hypothetical protein	6.25 / 10.33

ORF	ORF position bp	Amino acid length	Strand	% Identity with GenBank phage protein sequences by BlastP	Predicted function	pI/Mw (kDa)
ORF 95	72732-72854	40	+	100% (Podoviridae sp.[DAE75217.1])	hypothetical protein	4.20 / 4.24
ORF 96	72851-73108	86	+	98.84% (Klebsiella phage VLCpiP4a [UVX31021.1])	hypothetical protein	7.87 / 9.47

Table S2. Homologs in the KPYAP-1 Genome That Matched the KP8 Genome

KPYAP-1 ORF	Description and Accession Id of KP8 sequence	Max Score	Total Score	Query Cover	E value	Per. Ident	Acc. Len
1	No homology						
2	hypothetical protein HWB55_gp001 [Klebsiella phage KP8]YP_009837442.1	199	199	100%	1.00E-63	91.59%	107
3	No homology						
4	hypothetical protein HWB55_gp003 [Klebsiella phage KP8]YP_009837444.1	110	110	100%	1.00E-29	71.43%	70
5	RNA polymerase 1 subunit A [Klebsiella phage KP8]YP_009837446.1	328	32800%	100%	6.00E-113	95.73%	164
6	hypothetical protein HWB55_gp006 [Klebsiella phage KP8]YP_009837447.1	95.5	95.5	61%	3.00E-23	70.49%	65
7	hypothetical protein HWB55_gp007 [Klebsiella phage	120	120	99%	1.00E-32	54.46%	112

KPYAP-1 ORF	Description and Accession Id of KP8 sequence	Max Score	Total Score	Query Cover	E value	Per. Ident	Acc. Len
	KP8]YP_009837448.1						
8	putative helical domain containing protein [Klebsiella phage KP8]YP_009837449.1	122	122	100%	1.00E-34	96.83%	63
9	putative helical domain containing protein [Klebsiella phage KP8]YP_009837450.1	49.7	49.7	100%	1.00E-06	67.74%	31
10	hypothetical protein HWB55_gp011 [Klebsiella phage KP8]YP_009837452.1	119	119	98%	2.00E-33	90.62%	64
11	No homology						
12	hypothetical protein HWB55_gp012 [Klebsiella phage KP8]YP_009837453.1	124	124	100%	3.00E-35	84.51%	71

KPYAP-1 ORF	Description and Accession Id of KP8 sequence	Max Score	Total Score	Query Cover	E value	Per. Ident	Acc. Len
13	putative peptidoglycan binding domain comtaining protein [Klebsiella phage KP8]YP_009837454.1	196	196	100%	2.00E-62	93.14%	102
14	GTP-binding domain [Klebsiella phage KP8]YP_009837455.1	228	228	100%	7.00E-75	90.68%	118
15	No homology						
16	hypothetical protein HWB55_gp015 [Klebsiella phage KP8]YP_009837456.1	183	183	100%	8.00E-58	93.48%	92
17	antirepressor protein [Klebsiella phage KP8]YP_009837457.1	209	209	97%	6.00E-68	98.06%	104
18	DUF4326 domain-containing protein [Klebsiella phage KP8]YP_009837458.1	235	235	100%	3.00E-77	89.68%	127
19	No homology						

KPYAP-1 ORF	Description and Accession Id of KP8 sequence	Max Score	Total Score	Query Cover	E value	Per. Ident	Acc. Len
20	hypothetical protein HWB55_gp018 [Klebsiella phage KP8]YP_009837459.1	60.5	60.5	100%	5.00E-10	46.15%	62
21	ADP-ribosylglycohydrolase [Klebsiella phage KP8]YP_009837460.1	252	252	100%	4.00E-84	95.24%	126
22	hypothetical protein HWB55_gp020 [Klebsiella phage KP8]YP_009837461.1	53.9	53.9	44%	9.00E-07	62.50%	91
23	RNA polymerase RNAP1 subunit B [Klebsiella phage KP8]YP_009837463.1	556	556	100%	0	96.31%	271
24	hypothetical protein HWB55_gp023 [Klebsiella phage KP8]YP_009837464.1	190	190	100%	2.00E-60	93.88%	98
25	No homology						
26	RNA polymerase [Klebsiella phage KP8]YP_009837466.1	823	823	100%	0	97.77%	404

KPYAP-1 ORF	Description and Accession Id of KP8 sequence	Max Score	Total Score	Query Cover	E value	Per. Ident	Acc. Len
27	structural protein with Ig domain [Klebsiella phage KP8]YP_009837467.1	335	335	100%	2.00E-115	97.66%	171
28	hypothetical protein HWB55_gp028 [Klebsiella phage KP8]YP_009837469.1	128	128	100%	6.00E-37	95.45%	67
29	endonuclease [Klebsiella phage KP8]YP_009837470.1	238	238	100%	4.00E-79	97.41%	116
30	putative transmembrane helical domain containing protein [Klebsiella phage KP8]YP_009837471.1	100	100	98%	9.00E-26	73.13%	63
31	No significant similarity found						
32	putative 2 transmembrane helical domains containing protein [Klebsiella phage KP8]YP_009837472.1	120	120	100%	6.00E-34	95.08%	61

KPYAP-1 ORF	Description and Accession Id of KP8 sequence	Max Score	Total Score	Query Cover	E value	Per. Ident	Acc. Len
33	hypothetical protein HWB55_gp032 [Klebsiella phage KP8]YP_009837473.1	228	228	100%	5.00E-75	92.44%	119
34	hypothetical protein HWB55_gp033 [Klebsiella phage KP8]YP_009837474.1	239	239	100%	2.00E-79	97.44%	117
35	hypothetical protein HWB55_gp034 [Klebsiella phage KP8]YP_009837475.1	203	203	100%	5.00E-65	86.09%	112
36	ATPase [Klebsiella phage KP8]YP_00983747	726	726	100%	0	99.43%	350
37	HNH endonuclease [Klebsiella phage KP8]YP_009837477.1	798	798	100%	0	100.00%	383
38	dCTP deaminase [Klebsiella phage KP8]YP_009837478.1	359	359	100%	5.00E-125	98.86%	175

KPYAP-1 ORF	Description and Accession Id of KP8 sequence	Max Score	Total Score	Query Cover	E value	Per. Ident	Acc. Len
39	hypothetical protein HWB55_gp038 [Klebsiella phage KP8]YP_009837479.1	97.4	9740%	100%	1.00E-24	77.61%	67
40	hypothetical protein HWB55_gp039 [Klebsiella phage KP8]YP_009837480.1	162	162	100%	2.00E-49	78.64%	103
41	thymidilate synthase [Klebsiella phage KP8]YP_009837481.1	581	581	100%	0	91.05%	313
42	hypothetical protein HWB55_gp041 [Klebsiella phage KP8]YP_009837482.1	158	158	100%	2.00E-48	97.44%	78
43	hypothetical protein HWB55_gp042 [Klebsiella phage KP8]YP_009837483.1	172	172	100%	2.00E-53	82.86%	105
44	No homology						
45	RIIA lysis inhibitor [Klebsiella phage KP8]YP_009837485.1	1633	1633	100%	0	92.74%	840

KPYAP-1 ORF	Description and Accession Id of KP8 sequence	Max Score	Total Score	Query Cover	E value	Per. Ident	Acc. Len
46	RIIB lysis inhibitor [Klebsiella phage KP8]YP_009837486.1	1358	1358	100%	0	98.53%	678
47	hypothetical protein HWB55_gp046 [Klebsiella phage KP8]YP_009837487.1	269	269	100%	1.00E-90	98.47%	131
48	triphosphate pyrophosphohydrolase [Klebsiella phage KP8]YP_009837488.1	228	228	100%	1.00E-74	90.68%	134
49	DNA helicase [Klebsiella phage KP8]YP_009837489.1	902	902	100%	0	99.54%	435
50	hypothetical protein HWB55_gp049 [Klebsiella phage KP8]YP_009837490.1	367	367	100%	3.00E-128	98.86%	176
51	DNA polymerase [Klebsiella phage KP8]YP_009837491.1	1776	1776	100%	0	99.42%	860

KPYAP-1 ORF	Description and Accession Id of KP8 sequence	Max Score	Total Score	Query Cover	E value	Per. Ident	Acc. Len
52	hypothetical protein HWB55_gp052 [Klebsiella phage KP8]YP_009837493.1	195	195	100%	2.00E-62	95.96%	99
53	3'-phosphatase 5'-polynucleotide kinase [Klebsiella phage KP8]YP_009837494.1	209	209	100%	6.00E-67	74.47%	136
54	exonuclease [Klebsiella phage KP8]YP_009837495.1	669	669	100%	0	99.38%	323
55	DNA primase [Klebsiella phage KP8]YP_009837496.1	1498	1498	100%	0	99.86%	715
56	Sak4-like ssDNA annealing protein [Klebsiella phage KP8]YP_009837497.1	520	520	100%	0	100.00%	252
57	single strand DNA binding protein [Klebsiella phage KP8]YP_009837498.1	531	531	100%	0%	98.86%	264
58	holliday junction resolvase [Klebsiella phage KP8]YP_009837499.1	381	381	100%	3.00E-133	98.92%	185

KPYAP-1 ORF	Description and Accession Id of KP8 sequence	Max Score	Total Score	Query Cover	E value	Per. Ident	Acc. Len
59	hypothetical protein HWB55_gp059 [Klebsiella phage KP8]YP_009837500.1	187	187	100%	7.00E-58	90.34%	145
60	hypothetical protein HWB55_gp061 [Klebsiella phage KP8]YP_009837502.1	120	120	85%	8.00E-32	52.94%	125
61	hypothetical protein HWB55_gp062 [Klebsiella phage KP8]YP_009837503.1	162	162	100%	6.00E-50	96.30%	81
62	hypothetical protein HWB55_gp097 [Klebsiella phage KP8]YP_009837504.1	244	244	100%	2.00E-80	84.14%	140
63	hypothetical protein HWB55_gp095 [Klebsiella phage KP8]YP_009837506.1	333	333	100%	8.00E-115	95.73%	164
64	hypothetical protein HWB55_gp093 [Klebsiella phage	154	154	100%	9.00E-47	90.24%	82

KPYAP-1 ORF	Description and Accession Id of KP8 sequence	Max Score	Total Score	Query Cover	E value	Per. Ident	Acc. Len
	KP8]YP_009837508.1						
65	virion RNA polymerase [Klebsiella phage KP8]YP_009837509.1	7057	7057	100%	0	98.65%	3474
66	virion structural protein [Klebsiella phage KP8]YP_009837510.1	1288	1288	100%	0	99.08%	649
67	structural protein [Klebsiella phage KP8]YP_009837511.1	285	285	100%	2.00E-96	97.26%	146
68	putative 4 transmembrane helical domains containing protein [Klebsiella phage KP8]YP_009837512.1	1788	1788	100%	0	97.74%	886
69	virion structural protein [Klebsiella phage KP8]YP_009837513.1	554	554	100%	0	96.74%	276
70	hypothetical protein HWB55_gp087 [Klebsiella phage	417	417	100%	5.00E-147	98.05%	205

KPYAP-1 ORF	Description and Accession Id of KP8 sequence	Max Score	Total Score	Query Cover	E value	Per. Ident	Acc. Len
	KP8]YP_009837514.1						
71	major head protein [Klebsiella phage KP8]YP_009837515.1	818	818	100%	0	98.75%	401
72	tail length tape measure protein [Klebsiella phage KP8]YP_009837516.1	823	823	100%	0	98.78%	409
73	hypothetical protein HWB55_gp084 [Klebsiella phage KP8]YP_009837517.1	234	234	100%	3.00E-77	98.28%	116
74	portal protein [Klebsiella phage KP8]YP_009837518.1	1560	1560	100%	0	98.95%	761
75	Rz-like spanin [Klebsiella phage KP8]YP_009837519.1	328	328	99%	4.00E-113	98.79%	165
76	endolysin [Klebsiella phage KP8]YP_009837520.1	429	429	100%	3.00E-151	97.64%	212

KPYAP-1 ORF	Description and Accession Id of KP8 sequence	Max Score	Total Score	Query Cover	E value	Per. Ident	Acc. Len
77	holin [Klebsiella phage KP8]YP_009837521.1	179	179	100%	3.00E-56	98.86%	108
78	tail length tape-measure protein [Klebsiella phage KP8]YP_009837522.1	215	215	100%	4.00E-70	99.09%	110
79	No homology						
80	No homology						
81	No homology						
82	No homology						
83	No homology						
84	virion structural protein [Klebsiella phage KP8]YP_009837526.1	484	484	100%	2.00E-172	98.72%	235
85	large subunit terminase [Klebsiella phage KP8]YP_009837527.1	1097	1097	100%	0	99.06%	530
86	small terminase subunit [Klebsiella phage KP8]YP_009837528.1	468	468	100%	3.00E-166	99.13%	230

KPYAP-1 ORF	Description and Accession Id of KP8 sequence	Max Score	Total Score	Query Cover	E value	Per. Ident	Acc. Len
87	hypothetical protein HWB55_gp072 [Klebsiella phage KP8]YP_009837529.1	192	192	100%	4.00E-61	96.94%	98
88	hypothetical protein HWB55_gp071 [Klebsiella phage KP8]YP_009837530.1	226	226	100%	2.00E-74	97.27%	110
89	hypothetical protein HWB55_gp070 [Klebsiella phage KP8]YP_009837531.1	174	174	93%	1.00E-53	90.48%	110
90	hypothetical protein HWB55_gp069 [Klebsiella phage KP8]YP_009837532.1	192	192	100%	4.00E-61	83.33%	108
91	No homology						
92	hypothetical protein HWB55_gp068 [Klebsiella phage KP8]YP_009837533.1	41.2	41.2	100%	0.012	32.00%	61

KPYAP-1 ORF	Description and Accession Id of KP8 sequence	Max Score	Total Score	Query Cover	E value	Per. Ident	Acc. Len
93	hypothetical protein HWB55_gp067 [Klebsiella phage KP8]YP_009837534.1	60.8	60.8	93%	4.00E-10	51.67%	68
94	hypothetical protein HWB55_gp063 [Klebsiella phage KP8]YP_009837538.1	182	182	100%	1.00E-57	94.44%	90
95	No homology						
96	hypothetical protein HWB55_gp001 [Klebsiella phage KP8]YP_009837442.1	167	167	100%	1.00E-51	96.51%	107
