# Supplementary figures and images for: In Vitro and In Vivo Assessments of Newly Isolated N4-like Bacteriophage against ST45 K62 Capsular-Type Carbapenem-Resistant Klebsiella pneumoniae: vB_kpnP_KPYAP-1
Source: Int J Mol Sci. 2024 Sep 4;25(17):9595. doi: 10.3390/ijms25179595 (PMC11395603; doi:10.3390/ijms25179595)

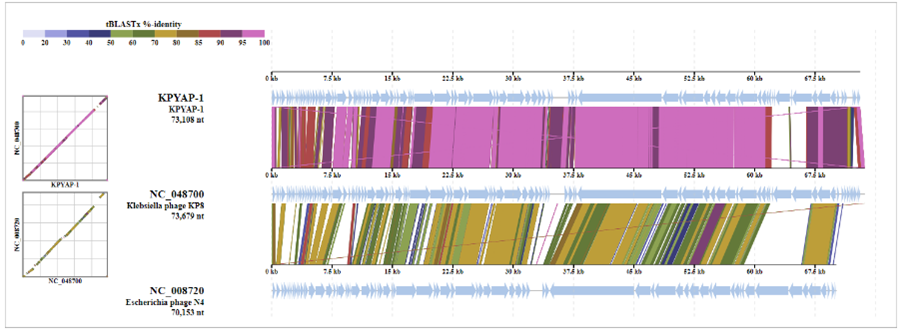

Supplement: Supplementary file 1 [file ijms-25-09595-s001.zip › Figure S1.tif]
